# Women’s perspectives on the acceptability of risk-based cervical cancer screening

**DOI:** 10.1186/s12885-024-13050-7

**Published:** 2024-10-25

**Authors:** Maali-Liina Remmel, Kadri Suija, Riina Raudne, Anna Tisler, Anda Ķīvīte-Urtāne, Mindaugas Stankūnas, Mari Nygård, Gunvor Aasbø, Laura Maļina, Anneli Uusküla

**Affiliations:** 1 Institute of Family Medicine and Public Health, Faculty of Medicine, University of Tartu, Ravila 19, Tartu, 50411 Estonia; 2 Institute of Public Health and Clinical Nutrition, Faculty of Health Sciences, University of Eastern Finland, Yliopistonrinne 3, Kuopio, FI-70211 Finland; 3 Institute of Public Health, Riga Stradiņš University, Dzirciema iela 16, Riga, LV-1007 Latvia; 4 Department of Health Management, Lithuanian University of Health Sciences, A Mickevičiaus g. 9, Kaunas, LT-44307 Lithuania; 5 Department of Research, Cancer Registry of Norway, P.O. box 5313, Oslo, Majorstuen Norway; 6 Department of Interdisciplinary Health Science, University of Oslo, Forskningsveien 3A, Oslo, 0373 Norway

**Keywords:** Cervical cancer screening, Risk-based screening, Acceptability of healthcare interventions, qualitative research

## Abstract

**Background:**

The increased knowledge of cervical cancer (CC) risk factors and suboptimal performance of present screening programs has generated interest in shifting from a universal screening approach to one based on individual risk assessment. To inform the future development of risk-based CC screening programs, it is crucial to gain insight into the factors influencing the acceptability of such approach among screening target group women. The aim of this study was to prospectively investigate the acceptability of risk-based CC screening and to identify potential barriers.

**Methods:**

In this qualitative study, one-to-one semi-structured interviews were conducted with a purposeful sample including women aged 30–65 years to explore women’s perspectives on the acceptability of risk-based CC screening. The study was conducted in Estonia, and interviews were conducted from March to September 2023. Potential participants were approached in person by a member of the study team or by their healthcare providers at primary care or gynaecology clinics. The interview guides were developed based on the concept of acceptability of healthcare interventions.

**Results:**

Twenty participants (mean age 44.5, SD = 8.6) with diverse backgrounds were interviewed. The seven components of acceptability (affective attitude, burden, ethicality, opportunity costs, perceived effectiveness, self-efficacy, and intervention coherence) were explored as key themes. Generally, women supported risk-based screening. However, we identified several factors that may compromise the acceptability of risk-based screening. The participants were reluctant to accept less intense screening for low-risk women and anticipated that if risk-based approach was implemented, more frequent testing would remain an option. Providing in-person clinician support was expected, requiring additional healthcare resources. Knowledge gaps in CC prevention highlighted the need for accessible information and education. Most women were unworried about sensitive data inclusion in risk score calculations. However, some participants were concerned about potential confidentiality breaches by healthcare workers.

**Conclusion:**

This study indicates that risk-based CC screening is acceptable, except for testing low-risk women less frequently. Our findings underscore the necessity for comprehensive understanding of the needs and concerns of the target group women for program development. Healthcare organizations are required to proactively address these needs by implementing comprehensive information dissemination and efficient communication approaches.

**Supplementary Information:**

The online version contains supplementary material available at 10.1186/s12885-024-13050-7.

## Introduction

The paradigm of cancer screening is undergoing transformation away from a uniform approach [1–5]. Universal (age, gender-based) screening has drawbacks, such as harms from overdiagnosis and inefficient use of health care resources [6]. Research on risk-based cancer screening includes predominantly studies on breast cancer, but also various other cancer types like colorectal, lung, prostate, ovarian, kidney cancer and melanoma have been studied [5, 7–13]. 

There is ongoing research interest to develop risk-based screening programs for cervical cancer (CC) [14–21] as its ethiopathogenesis is well-defined and extensively researched with persistent high-risk HPV (*human papilloma virus*) infection and malignant transformation being central in the ethiopathogenesis of cervical cancer. Cofactors increasing cervical cancer risk include younger age of first sexual intercourse, higher number of lifetime sexual partners, previous sexually transmitted diseases, immunosupression, smoking, prolonged oral contraceptive use, higher parity, recurrent pregrancy loss and lower socioeconomic status [22–27]. 

The shift to risk-based approach is further prompted by the ineffectiveness of existing programs, attributed to factors such as low or decreasing coverage rates in many regions, disparities affecting minorities and vulnerable populations, and the concomitant opportunistic testing [28–31]. 

In Estonia, the estimated age-standardized incidence of cervical cancer was 11,8 per 100 000 in 2022 [32]. An organized cervical cancer screening program using cytology-based Papanicolaou (PAP) testing every 5 years was introduced in 2006, targeting women aged 30 to 55 years. Cervical cancer screening attendance has been low and has not exceeded 50% [33]. ^,^ [34] In parallel, opportunistic screening is extremely prevalent, constituting 75% of all annual cytology tests performed in Estonia [35]. In 2021, a 5-yearly primary HPV screening targeting women aged 30 to 65 years was implemented in Estonia.

The transition to risk-based approach is challenging, as it entails altering current practices and requires engagement and acceptance from screening participants and healthcare providers.

Acceptability is becoming one of the key considerations in the process of designing, evaluating, and implementing healthcare interventions [36]. Yet, there is a notable absence of research specifically focused on the acceptability of risk stratification in cervical cancer screening (CCS) [5]. 

Therefore, in this study, we explored the prospective acceptability of risk-based CCS to inform program development that aligns with women’s needs and preferences.

## Methods

### Design

We used a qualitative descriptive approach [37] involving semi-structured interviews, aimed to investigate the acceptability of risk-based CCS among women living in Estonia. This study followed Consolidated Criteria for Reporting Qualitative Research (COREQ) [38]. 

### Setting and participants

The study was conducted among CC screening target group women in Estonia.

Purposive sampling, also known as judgment or selective sampling was used, which involves the researcher using their own discretion to select participants from the population for the study. In order to capture unique and diverse variations while identifying common patterns across them, we opted for maximum variation sampling among the various purposeful sampling designs available [39]. Background characteristics ensuring high participant variation were chosen based on literature related to engagement and experiences in cervical cancer screening [40–42]. This included adults of various ages, education levels, ethnicities, parities, screening histories, and HPV test results, as these factors could potentially influence risk perception.

Potential participants were invited personally by a member of the study team or by their healthcare providers at primary care or gynaecology clinics.

Of the 50 women invited to participate, 20 enrolled. Participants selected a convenient interview time and mode (phone, web platform or on-site). Written informed consent was obtained from all participants prior to the interviews.

### Risk-based cervical cancer screening acceptability

**Fig. 1:**
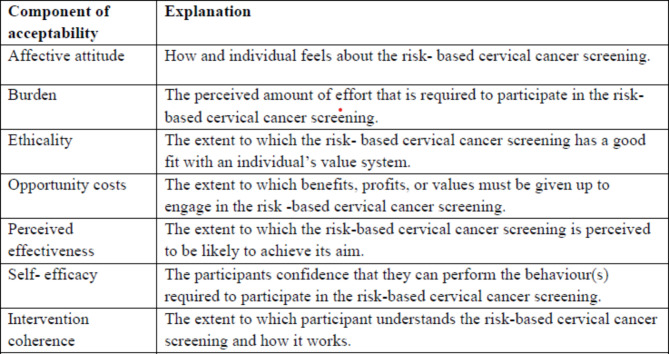
The components of acceptability of risk-based cervical cancer screening. Explanations adapted from Sekhon et al. [36]

Women in the CCS target group can form opinions about the prospective acceptability of an intervention before experiencing it. We use the definition of the acceptability of healthcare interventions developed by Sekhon et al. as our foundational framework: a multi-faceted construct that reflects the extent to which people delivering or receiving an intervention consider it to be appropriate, based on anticipated or experienced cognitive and emotional responses to the intervention consisting of seven constructs: affective attitude, burden, perceived effectiveness, ethicality, intervention coherence, opportunity costs, and self-efficacy (see Fig. 1) [36]. 

### Data collection

The data was collected by in-depth semi-structured interviews.

The interview guide was developed collaboratively (AK-U, GA, KS, MS, AT, MN, LM, AU, MLR) based on the literature regarding the Theoretical Framework of Acceptability (TFA) [36, 43, 44]. The guide was developed in English and translated and adapted to Estonian. To facilitate a clearer comprehension of risk-based screening, we created a short introductory video that was presented to the participants preceding the interview. The video conveyed information about CC risk factors, the setup of the current screening program, and the principles of risk-based screening, along with realistic examples of low- and high-risk scenarios.

Two hypothetical scenarios were presented, each illustrating different levels of cervical cancer (CC) risk. In the low-risk scenario, a 30-year-old woman with HPV-negative status and no history of cervical abnormalities or pregnancies was advised to undergo screening every 10 years due to her low-risk status [45, 46]. The high-risk scenario involved a 54-year-old woman with a positive high risk HPV test, history of cervical abnormalities, three pregnancies, and one abortion. She was advised annual screening due to her elevated risk.

The interview guide is provided as an additional file (see Additional file 1).

The interviews were conducted by four female researchers (MLR, AT, AU or KS) - three medical doctors and a public health researcher, all of whom had previous experience in conducting qualitative interviews.

Most interviews were performed via web-based platform (Teams), some on-site (*n* = 2) or by phone (*n* = 2). The interviews were digitally recorded, transcribed verbatim, and de-identified. We did not carry out any repeat interviews. The participants had possibility to review and comment the transcripts, one used it, and confirmed that no modifications were needed as the as the recorded material accurately reflected her sentiments.

Twelve interviews were performed in Estonian and eight in Russian (by a researcher who is a native Russian speaker). Interviews were conducted between March and September 2023.

### Data analysis

A qualitative descriptive approach, employing iterative inductive and deductive thematic analysis was used [47]. 

The process began with independent line-by-line open coding of 7 of the transcripts by four analysts (MLR, KS, AT, RR) which informed the development of a codebook. Subsequently, the entire data was coded, refining, and adding codes throughout the process [47]. The codes were sorted into groups sharing similar content to develop subthemes. The subthemes were constantly compared and cross-referenced between transcripts. Subsequently, the subthemes were grouped under the seven constructs of TFA considered as key themes [36]. The final sample size (*n* = 20) was based on data saturation (the point at which no new information or themes were observed in the data) assessed through theme discussion among the authors [48]. 

We had regular meetings with the team, which included clinicians (MLR, KS, AU), a non-clinician qualitative researcher (RR) and a public health researcher (AT) to discuss the interpretations. All researchers involved agreed on the derived subthemes and their grouping under the key themes.

To reduce the impact of personal biases, we double-coded the interviews (two researchers individually analyzed each interview, and then engaged in a dialogue to reconcile any significant discrepancies that may have arisen) and aimed to keep the analysis close to the participants’ statements. We also strived to ensure high reflexivity (i.e. self-examination of how researchers position, background, pre-existing beliefs and shared experiences with the study partcipants can affect the interviewing and analysis) [49, 50]. Our team of researchers consisted of women aged 33–56, with differing professional backgrounds of clinical medicine, public health, social sciences. One of the researchers was a native russian-speaker and she conducted the interviews with the russian speaking women. The regular discussions of the interpretations with our team of researchers supported reflexivity and helped to reduce the unconcious bias.

We used NVivo software version 12 (QSR International) to analyze the data.

## Results

20 interviews were conducted with participants whose mean age was 44.5 (SD = 8.6) years (Table 1). Ten had higher, 5 secondary, 2 vocational and 3 primary education. Eight were Russian speakers and 12 spoke Estonian. The number of births varied 0–4. Twelve women had participated in national CCS. It is important to acknowledge that this does not autmatically imply that non-participants had not undergone a PAP-smear or HPV-test at their doctor’s office apart form the formal screening program. The interviews lasted from 18 to 61 (mean of 33.1) minutes.

**Table 1:** Characteristics of the participants

Number of the interview	Year of birth	Education	Number of children	Language	HPV*	Has ever participated in national cervical cancer screening
1.	1960	Higher	1	Estonian	no	yes
2.	1970	Vocational	1	Estonian	no	yes
3.	1970	Vocational	3	Estonian	yes	yes
4.	1970	Secondary	2	Estonian	no	no
5.	1970	Secondary	2	Russian	NA	yes
6.	1973	Higher	3	Estonian	no	yes
7.	1974	Primary	1	Estonian	no	yes
8.	1977	Secondary	2	Russian	yes	yes
9.	1978	Higher	1	Estonian	no	no
10.	1979	Higher	4	Russian	NA	no
11.	1979	Primary	4	Estonian	no	yes
12.	1981	Higher	0	Estonian	yes	yes
13.	1982	Higher	2	Estonian	yes	no
14.	1983	Higher	3	Russian	NA	yes
15.	1984	Higher	0	Russian	NA	no
16.	1986	Secondary	1	Russian	NA	no
17.	1988	Primary	1	Estonian	no	yes
18.	1991	Higher	3	Estonian	no	yes
19.	1991	Higher	1	Russian	NA	no
20.	1993	Secondary	1	Russian	NA	no

*Had been tested positive for HPV in previous 12 months (yes/no/NA- not available)

Fifteen subthemes were identified and classified under the seven key themes of TFA (subthemes are presented in a supplementary table in Additional file 2). The following sections describe the results of each key theme.

### Affective attitude

The concept of risk-based screening was met with enthusiasm by most women and positive feelings were expressed towards personal approach as such, as one woman stated, “It appreciates you as an individual and it is personally analysed, what approach I would need and what someone else would need. It is better than measuring everyone by the same means.” (Participant 7).

However, introducing the idea that women with lower risk would be given a recommendation of a longer screening interval - once every 10 years, yielded a strong negative response like “It´s totally unacceptable” (P 4) except for one participant who said that such an interval is suitable. Multiple women expressed an interest in seeking alternative avenues if needed, such as more frequent testing in a private clinic. One woman responded, “Even if it (the risk) is low, I want to be tested. I would find a way and reasons to have it (test) done.” (P 11).

On the other hand, testing high-risk women more often was perceived as important by all the participants. In one woman’s words, “Definitely, this is one of the ways to diagnose cancer in a stage where you can do something about it.” (P 18).

### Burden

A hypothetical scenario describing a low risk was predominantly received with relief. However, the perceived need for frequent testing remained essential for maintaining reassurance, with one participant stating,” If you can go every year, it gives you peace and feeling of not needing to worry.” (P 17).

Information about elevated risk caused anxiety, but the women also saw this as beneficial, allowing proactive measures, such as lifestyle changes and more frequent screening. The changes in lifestyle that women referred to included general measures like a healthy diet and exercise, as well as avoiding harmful habits like smoking. However, they did not mention changes in sexual behavior.

One woman with a previous high-risk result of antenatal screening for genetic disorders said, “I would not want to know.’ I’ve had that experience and it’s psychologically hard to bear.” (P 9).

Most women did not find risk-based CCS physically burdensome, though some found it uncomfortable. However, as one participant said, “It is unpleasant, but it needs to be done.” (P 7).

Several participants raised concerns about vulnerable groups (e.g., less educated, rural dwellers), noting that risk-based screening could be cognitively demanding and therefore reduce adherence. One woman said: “… these people with numerous children and without education, who have had many intercourses, abortions, these are the ones in danger, the information may not get through to them and they don’t know what to do with it.” (P 1) These statements indicated a perceived distinction between “other women” and themselves. As one interviewee put it: “It would not be an issue for me, but I know many women, especially people living in the countryside, for example, or older women not using the internet to whom the information may not reach as well, my mother would be an example.”(P 10).

### Ethicality

Most women felt that varying screening intervals for different risk levels was fair, as one participant rationalized, “People do not go to doctors with the same intervals anyway.” (P 17) However, one individual thought that it was unfair to differentiate screening based on risk stating “This would not be fair. I think both high and low risk women should be annually tested. It is important to keep an eye on it.” (P 6).

The use of personal health data was generally not a concern, as one participant stated, “It’s my health. Why can’t a doctor use my data to draw conclusions? It’s necessary.” (P 19) Considerable number of women were willing to provide additional personal health info, like the number of sexual partners, age of first sexual encounter, number of pregnancies and abortions, and smoking history. However, some were hesitant to share information about the aforementioned risk score components: “I’d like to hide it, like the number of sexual partners.” (P 5).

Some interviewees were concerned about confidentiality and hoped it would not be breached, “It is a complicated issue … Estonia is a small country … my friend is a nurse, and she came to discuss something about our mutual friend, something she was not supposed to disclose.” (P 6).

### Opportunity costs

For most women the comprehension of the risk seemed to be an empowering factor that outweighs the related costs like possible anxiety, time spent on testing, discomfort of the procedure or allowing the use of personal information. As one interviewee expressed, “High risk would make me scared, but if I was tested every year, I would not see a cause for concern.” (P 4) One woman said, “My health is a very important value to me, I don’t see any reason why I couldn’t find the one or two hours once a year for testing.” (P 10) and another „There are no side effects to frequent testing. The more we check, the better, because it’s better to prevent than to treat. “ (P 14).

### Perceived effectiveness

Most women deemed risk-based screening rational and beneficial. One participant stated, “it seems like a logical step forward.” (P 2) Benefits listed included cancer risk reduction, early diagnosis, anxiety relief, and increased awareness. Only a few women underscored the potential of risk-based screening to decrease the burden on the healthcare system.

However, women questioned the efficacy of personalised screening in cases of longer interval recommendations. They raised doubts about the durability of the risk score, as behaviour can change over a decade. As one woman said, “In 10 years, a woman can start drinking and smoking … data must be constantly updated during that time, she could have had an abortion, she could have more partners.” (P 8).

Many interviewees suggested that personalized screening and invitations communicating individually calculated risk would encourage more women to participate in the program: “Individual approach would be more effective. An anonymous invite makes you feel like a drop in the ocean, like it doesn’t matter if you go or not.” (P 10).

Several participants expressed the hope and belief that a sufficient effort by the medical system will be undertaken to ensure the efficacy and evidence base of a new screening program. They expected the program to be introduced cautiously, enabling them to confidently trust its ability to prevent a late diagnosis of a serious disease, as one woman stated, ” All steps should be taken to ensure that this is safe.” (P 18).

### Self-efficacy

The interviews revealed several conditions necessary for women to feel confident that participation in risk-based CCS could be accomplishable.

Comprehensive information and clear explanations of results were deemed important. Women expected extra information to help them understand their risk, what steps to take and hoped this information would be in simple language and delivered by their own gynaecologists and general practicioners, especially in high-risk cases. As a participant stated, “I would want to know it all (information on test results), I have been going to this doctor (gynaecologist) for my whole life, I would trust her.” (P 3).

Several women indicated a desire for the doctor to take the lead in screening, with one saying,” If we had this kind of screening, an individual trusted doctor should lead it. I don’t want more people involved.” (P 4) Most people felt that despite trusting their doctor’s advice, women should have the freedom to choose their own screening intervals.

Women emphasized the need for experienced healthcare personnel to conduct the tests: “I don’t want to be a guinea pig for someone who gives me a false result and then I get a cancer diagnosis in five months.” (P 8).

A proportion of interviewees expressed low enthusiasm for preventive efforts and CCS when no symptoms present. One participant said, “I’m not really into this. I’m lazy and don’t think about the future when I’m symptom-free.” (P 14).

### Intervention coherence

The participants vested authority in evidence-based risk profiling and personal counselling by doctors. However, the role of the organized screening program in ensuring efficacy was not acknowledged and most women did not distinguish between opportunistic screening and participation in the national screening program. The general understanding of the framework of organised cervical cancer screening was low. As one woman answered, “Yes, I have participated in screening, but I did it at my doctors.” (P 4).

Interviews revealed gaps in knowledge regarding CC and its prevention. Many women incorrectly believed CC would always have early symptoms and could be self-monitored. One woman said, “Screening is important because not everyone is as aware of their body as I am.” (P 7) Multiple women saw frequent testing as beneficial only and were not aware of any drawbacks as one participant stated:” There are no harms to frequent testing.” (P3).

Women acknowledged the need for more information on CC prevention, “If I have a high risk, what can I do to prevent cancer? If I have it all (cervix) removed, will it completely prevent it? What are the signs that something is wrong? Does vaginal dryness indicate cervical cancer? What should I monitor regarding my partner? How does hormonal contraception affect it all?” (P 6).

## Discussion

Cervical cancer screening has been the cornerstone of cancer prevention, being the first and most effective of cancer screening programs [6, 51, 52]. Due to the historical success, ingrained practices, and patient trust, changing the universal paradigm in CC screening might be particularly challenging.

This is the first study to our knowledge to examine women’s acceptance of risk-based CCS. Some findings align with the existing literature on the acceptability of risk-based cancer screening, such as the generally positive attitude towards personalized approach, but also hesistancy to accept less frequent testing in low-risk cases [5, 53–55]. However, in our study, women were not only hesitant, but also willing to seek alternative avenues to continue frequent testing (varying from annually to every 5 years), seeking a sense of security. During the long Soviet occupation in Estonia, women were encouraged to have yearly PAP tests at their gynecologists´. This tradition of annual testing persists among both women and gynecologists and is challenging to relinquish, even though this opportunistic screening strategy in the absence of an organized and comprehensive screening program did not result in a decreased CC incidence or mortality [56]. 

Women recognized the importance of clearly communicating any alterations to the existing CCS model to the intended audience. Previous studies have emphasized the importance of raising awareness and providing accessible information [5, 55]. In addition, we identified several misconceptions related to CC prevention and screening. One prevalent misconception was the belief that CC exhibits early symptoms, leading some women to consider self-monitoring as a viable screening option. Women often perceived opportunistic testing and participation in national screening programs as interchangeable. However, extensive opportunistic screening can lead to inconsistent follow-up and compromised engagement over the years, thereby reducing its effectiveness [57]. Previous research has shown that overscreening resulting in false positive screening test results and conqurrent diagnostic and excisional treatments of potentially self-limiting lesions can cause both psychological problems like anxiety and physical health issues like pain, bleeding, discomfort and adverse obstertric outcomes [58–62]. Among the women interviewed, awareness of possible harms of overscreening was lacking.

Therefore, education and increasing health literacy among the screening population plays a crucial role in implementing risk-based approach. Women need to comprehend the reasoning behind the risk categories and the accuracy of the estimations. Low-risk individuals may benefit from clear guidance on the importance of regular screenings and the potential risks associated with delaying or forgoing them. On the other hand, high-risk women might require more detailed information about additional preventive measures or treatment options available to them. Providing tailored information specific to each risk group can empower individuals to make informed decisions about their cervical health and contribute to more effective screening strategies overall.

Our results indicate high demand for face-to-face patient consultations and support regarding women’s concerns and needs regarding individualized CC screening. The inclination to seek alternatives to continue frequent testing despite low risk and the need for counselling by doctors poses the challenge of risk-based screening potentially increasing healthcare demand instead of alleviating it.

Although age- and sex-based restrictions in screening are widely accepted, introducing additional eligibility criteria poses new ethical dilemmas. These include the need to prevent unintended discrimination and avoid reinforcing existing inequalities during the risk-assessment process. Incorporating both modifiable and non-modifiable risk factors may raise concerns, especially when they intersect with other socially significant categories, such as ethnicity, disability, or social class. These aspects must be considered to ensure accessible and fair screening. A strength of this study was the inclusion of Russian speaking women, a vulnerable minority in Estonia with lower participation in CCS requiring potential focused attention [41]. 

CC risk estimation differs from other cancer types as potentially sensitive information like details of sexual history are required for a more accurate calculation. Therefore, as our study highlighted, there needs to be high effort to ensure confidentiality.

The introduction of HPV-vaccination underscores the importance of investigating efficient alternatives to one-size-fits-all cervical cancer screening as when cohorts of women who have been vaccinated against HPV reach the age for cervical cancer screening, implementing risk-based screening becomes imperative. Furthermore, the rapidly growing digital health information highlights the potential to develop a more personalized risk-based screening approach taking into acoount the sociodemographic, lifestyle, and health-related factors in addition to vaccination status.

Reducing the burden of CC is a global priority [63] that requires innovative yet acceptable approaches within the context of risk-based screening programs, underscoring the importance of this study and further research on the topic in wider contexts.

### Limitations

This study had limitations. Women willing to participate might hold different views from those not enrolled. As this is a qualitative study, the findings cannot be extended to wider populations or to different healthcare contexts. However, we believe that the findings still contribute to the understanding of the perspectives of women to be screened.

It is acknowledged that prospective and retrospective acceptability may differ. However, the assessment of anticipated acceptability is essential, as it can highlight which aspects of the intervention could be modified to increase acceptability and thus encourage greater participation [36, 64]. 

## Conclusion

The targeted approach in cervical cancer screening has the potential to enhance program effectiveness, optimize resource allocation, and improve prevention and management outcomes.

The findings of this study suggest a favorable perspective on risk-based cervical cancer screening among the screening target group. Women’s strong approval is not just pivotal but imperative for the future shift to risk-based approach.

Yet, this transformation demands meticulous planning to address the needs of the women and healthcare providers effectively. A patient approach, allowing ample time to navigate and conquer obstacles that may arise is essential.

## Electronic supplementary material

Below is the link to the electronic supplementary material.

Supplementary Material 1

Supplementary Material 2

## Data Availability

The datasets used and/or analysed during the current study are available from the corresponding author on reasonable request.

## Abbreviations

CC: cervical cancer
CCS: cervical cancer screening
HPV: human papilloma virus
PAP-test: Papanicolaou test
