# Supplementary material for: Women’s perspectives on the acceptability of risk-based cervical cancer screening
Source: BMC Cancer. 2024 Oct 25;24:1314. doi: 10.1186/s12885-024-13050-7 (PMC11515292; doi:10.1186/s12885-024-13050-7)
Supplement: Supplementary file 1 — Supplementary Material 1 [file 12885_2024_13050_MOESM1_ESM.docx]

**Supplementary content 1**

Remmel M-L, Suija K et al. Women’s perspectives on the acceptability of risk-based cervical cancer screening. A qualitative study in Estonia.

**Semi-structured interview guide to assess the acceptability of risk-based cervical cancer screening.**

| **Components** | **Questions** |
| --- | --- |
| **Initial questions** | The interviewer introduces themselves (name, position) and confirms that the consent form has been signed by the interviewee, and mentions that the conversation will be recorded. They validate that this is acceptable for the interviewee. The interviewer proceeds with introductory questions.   - What is your year of birth? - Where do you live? - Do you have children? - What is your education? - What do you do for living? - Have you ever participated in the cervical cancer screening? - When was the last time you were screened? |
| **Background information on risk-based cervical cancer screening**  A video explaining the concept of risk-based cervical cancer will be shown to the interviewee (or the script read in case of an interview conducted by phone). The script of the video is as follows:  „Cervical cancer develops when there is uncontrolled growth of abnormal cells in the cervix. Almost all cases of cervical cancer are caused by the sexually transmitted human papillomavirus (HPV). Cervical cancer can be prevented through vaccination and detected by screening. If it is detected early, it can often be cured. It is known that certain types of HPV are more likely to cause cervical cancer than others, and these are referred to as high-risk HPV subtypes.  The risk of developing cervical cancer is higher if a woman smokes or has been a passive smoker, has used hormonal contraceptives for a long time, or has a weakened immune system, for example due to the use of immunosuppressive medications or HIV-infection. Other factors that increase the risk include age, numerous sexual partners, and having had more than 5 pregnancies or abortions. The risk is also higher if there have been abnormalities in previous cervical screenings.  There is a growing exploration of the possibilities of moving towards a cervical cancer screening arrangement that takes into account an individual's specific risk for cervical cancer. This approach is called risk-based screening.  Currently, every woman in Estonia aged 30-65 is invited to participate in cervical cancer screening every five years, which involves testing for the cancer-causing HPV virus. This approach, testing all women in a certain age group, is referred to as the "one-size-fits-all" approach.  If specific information about each woman (age, HPV vaccination, previous screening results, number of pregnancies, abortions etc.) is collected from national registries and entered into a risk calculator, it is possible to calculate an individual's specific risk of developing cervical cancer. Based on this, each woman can receive a more personalized recommendation for how often she should participate in screening.  This approach is not currently used in Estonia.  To illustrate risk-based screening, I will present two examples:  **Story 1.**  Laura is 30 years old. She was informed that her risk of developing cervical cancer is lower than average because she is young, has no abnormalities in previous screenings, and has not been pregnant. Therefore, it is recommended for her to reduce the frequency of screening tests and have them done every 10 years instead of every 5 years.  **Story 2.**  Sirje is 54 years old. She was informed that her risk of developing cervical cancer is high because she is over 50, has previously had a positive HPV test (with a high-risk virus type), has been diagnosed with changes in cervical cells, and has had three pregnancies, one of which ended in an abortion. The recommendation is to increase the frequency of screening tests and have them done annually instead of every 5 years. “ | |
| **Affective attitude** | - - What do you think of the idea of risk-based screening?   - What would you like about this approach?   - What would you not like about this approach? - Let's imagine for a moment that you are Laura, who found out that her cancer risk is low.   - What would be your first thought in connection with this?   - If you were given information that you have a low risk of developing cervical cancer and were advised to undergo cervical cancer screening every 10 years (instead of once every 5 years), do you think such a testing frequency is appropriate, considering your low risk?   - What concerns, fears, or questions might arise in such a situation? Why?   - Do you think people would trust the recommendation that less frequent testing may be just as effective as the currently used testing every 5 years? - Let's imagine for a moment that you are Sirje, who was informed that she has a high cancer risk.   - What would be your first thought in connection with this?   - If you were found to have a high risk of cervical cancer and were advised to undergo screening once a year (instead of e every 5 years), do you think such a testing frequency is appropriate, considering your high risk?   - What concerns, fears, or questions might arise in such a situation? Why? - Would information about a high risk make screening more important for you? |
| **Burden** | - - If risk-based screening was to be implemented in Estonia, what information and support would you need to decide whether the new and personalized cervical cancer screening frequency recommendation is acceptable for you? (clarification if needed: support could include counseling, practical assistance with logistics, etc.)   - Would you like to know the magnitude of your risk (e.g. low or high) of developing cervical cancer? Please briefly explain your answer. |
| **Ethicality** | - Do you think it would be fair that each woman would be given an individual recommendation on how often she should undergo cervical cancer screening? - How would you feel if someone you know was screened at a different frequency than you? - What do you think about the use of your personal data for calculating risk?   - Would this help you decide whether to participate in screening or not? - Would you like a more detailed risk calculation and be willing to provide additional information for that purpose, such as the number of sexual partners and smoking habits, by entering them into a risk calculator? - In rare cases, the calculated risk based on your health registry data may be higher than the actual risk (a false positive result). In such case, you would be recommended screening (HPV testing) too frequently. In some cases, the opposite may occur and the calculated risk based on your health registry data may be too low (a false negative result). In this case, you would participate in screening too infrequently, e.g., once every 10 years, and early changes or cancer may be detected too late.   - What thoughts and feelings does the potential inaccuracy of risk assessment evoke in you?   - What do you think - how likely are errors in calculating cancer risk? - Risk-based screening can be highly beneficial for those with high risk, ensuring they are tested frequently and receive timely treatment if needed. However, in most cases, women with a high estimated risk do not develop cervical cancer. In such cases, frequent testing may be seen as excessive intervention and not beneficial to the individual. Women with an estimated low risk, tested once every 10 years, do not develop cervical cancer. Therefore, one could argue that they do not benefit from screening.   - What thoughts and feelings do these aspects evoke in you? |
|  |  |
| **Intervention coherence** | - Compared to other women of the same age, how high do you consider your risk of developing cervical cancer in the next 10 years?   - What do you think is the likelihood that at some point in the next 5-10 years you will develop cervical cancer? How about over your lifetime? - Do you feel it is clear to you why risk-based screening might be beneficial? Explain briefly. - Do you agree that women with a higher risk of cervical cancer should be tested more frequently? Why? - Do you agree that women with a lower risk of cervical cancer should be tested less frequently? Why? |
| **Opportunity costs** | - What impact would learning about your cancer risk have on your daily life in the case of low risk?   - What about in the case of high risk? - Would knowing the risk make you change your lifestyle, such as health and sexual habits? - How much would you be willing to invest your time, attention, and money to find out your individual cervical cancer risk and its meaning? - For some women, cervical sample collection may be uncomfortable, and it also requires time and the opportunity to get tested. If a woman is found to have a high risk, she may be advised to provide cervical samples for screening much more frequently than before. What thoughts and feelings does the possible additional effort associated with more frequent testing evoke in you? |
|  |  |
| **Perceived effectiveness** | - Would you agree that participating in risk-based screening reduces the risk of cervical cancer? Explain briefly. - Which strategy would you prefer - the current "invitation to everyone every 5 years" or individual risk-based screening? Why? |
| **Self-efficacy** | - How confident would you feel participating in risk-based screening? - Would you follow the recommended frequency for personal screening? Why? - Would knowing your personal risk affect your decision to undergo testing?   Imagine discussing risk-based screening and testing frequency with your doctor. What would you like to ask and discuss with them? |
| **Final questions** | - Would you trust the estimated cervical cancer risk calculation provided for you by the Estonian national healthcare system? Why? - Would you trust that the Estonian national registries contain accurate information about you (for example your age, HPV test results, number of pregnancies, abortions etc)? Why? - How confident are you that healthcare workers would handle your data confidentially? - What would be your preference for receiving your individual risk information- for example in person, over the phone, by letter, or reading it from your digital health record? - What additional information, besides individual risk and screening frequency recommendations, should be provided to women regarding risk-based screening? (e.g. additional information on a website, phone number/email address to contact for additional questions, who to call/write to ask for more information, such as what does my risk actually mean?) - Is there anything else you would like to address regarding risk-based screening that has not been mentioned in the interview?   Thank you very much for the interview. |
