# Supplementary material for: Women’s perspectives on the acceptability of risk-based cervical cancer screening
Source: BMC Cancer. 2024 Oct 25;24:1314. doi: 10.1186/s12885-024-13050-7 (PMC11515292; doi:10.1186/s12885-024-13050-7)
Supplement: Supplementary file 2 — Supplementary Material 2 [file 12885_2024_13050_MOESM2_ESM.docx]

**Supplementary content 2**

Remmel M-L, Suija K et al. Women’s perspectives on the acceptability of risk-based cervical cancer screening. A qualitative study in Estonia.

**Key themes and subthemes**

| **Theme** | **Description** |
| --- | --- |
| **Key theme 1. Affective attitude** | |
| Subtheme |  |
| 1 | General attitude |
| 2 | Screening intervals |
| **Key theme 2. Burden** | |
| Subtheme |  |
| 3 | Emotional burden |
| 4 | Physical burden |
| 5 | More vulnerable groups |
| **Key theme 3. Ethicality** | |
| Subtheme | |
| 6 | Personal data and confidentiality |
| 7 | Fairness |
| 8 | Value of feeling secure |
| **Key theme 4. Opportunity costs** | |
| Subtheme | |
| 9 | Benefits and costs |
| **Key theme 5. Perceived effectiveness** | |
| Subtheme | |
| 10 | Advantages |
| 11 | Adequacy of calculations and recommendations |
| **Key theme 6. Self-efficacy** | |
| Subtheme | |
| 12 | Prerequisites for acceptance |
| 13 | Participation in risk assessment |
| **Key theme 7. Intervention coherence** | |
| Subtheme | |
| 14 | Understanding the rationale |
| 15 | Knowledge and education |
